# Mechanisms That Modulate and Diversify BDNF Functions: Implications for Hippocampal Synaptic Plasticity

**DOI:** 10.3389/fncel.2019.00135

**Published:** 2019-04-09

**Authors:** Ana Paula De Vincenti, Antonella S. Ríos, Gustavo Paratcha, Fernanda Ledda

**Affiliations:** ^1^División de Neurociencia Molecular y Celular, Instituto de Biología Celular y Neurociencias, Universidad de Buenos Aires, CONICET, Buenos Aires, Argentina; ^2^Fundación Instituto Leloir, Instituto de Investigaciones Bioquímicas de Buenos Aires, CONICET, Buenos Aires, Argentina

**Keywords:** BDNF, Pro-BDNF, TrkB, hippocampus, synaptic plasticity

## Abstract

Brain-derived neurotrophic factor (BDNF) is a neurotrophin that has pleiotropic effects on neuronal morphology and synaptic plasticity that underlie hippocampal circuit development and cognition. Recent advances established that BDNF function is controlled and diversified by molecular and cellular mechanisms including trafficking and subcellular compartmentalization of different *Bdnf* mRNA species, pre- vs. postsynaptic release of BDNF, control of BDNF signaling by tropomyosin receptor kinase B (TrkB) receptor interactors and conversion of pro-BDNF to mature BDNF and BDNF-propeptide. Defects in these regulatory mechanisms affect dendritic spine formation and morphology of pyramidal neurons as well as synaptic integration of newborn granule cells (GCs) into preexisting circuits of mature hippocampus, compromising the cognitive function. Here, we review recent findings describing novel dynamic mechanisms that diversify and locally control the function of BDNF in hippocampal neurons.

## Introduction

The correct function of the nervous system depends on the proper establishment of the synaptic contacts achieved by neurons. For this, developing neurons must acquire a correct morphology that allows the formation of neuronal circuits. Alterations in neuronal morphology have been associated with different neuropathological conditions, characterized by cognitive defects (Forrest et al., 2018). Neuronal architecture is regulated by intrinsic and extrinsic factors, among which are neurotrophins (Park and Poo, 2013). Within this family, brain-derived neurotrophic factor (BDNF) is highly expressed in the hippocampus where it supports a variety of functions including regulation of neuronal morphology and synaptic plasticity by binding to the high-affinity receptor tyrosine kinase, tropomyosin receptor kinase B (TrkB; Leal et al., 2015).

In the hippocampus, the most prominent type of cells that determine the tri-synaptic circuitry are the pyramidal neurons that form the pyramidal layer of CA1 and CA3 regions, and the granule cells (GCs) of the dentate gyrus. Hippocampal pyramidal and dentate GCs express both TrkB and BDNF (Drake et al., 1999), and there is a large body of evidence indicating that BDNF is a relevant modulator of structural and functional synaptic plasticity in these type of excitatory neurons (Gonzalez et al., 2016; von Bohlen Und Halbach and von Bohlen Und Halbach, 2018). Although TrkB is also expressed in hippocampal GABAergic interneurons and important effects of BDNF on these inhibitory neurons have been reported (Porcher et al., 2018), this topic will not be discussed in the present revision.

**Figure 1:**
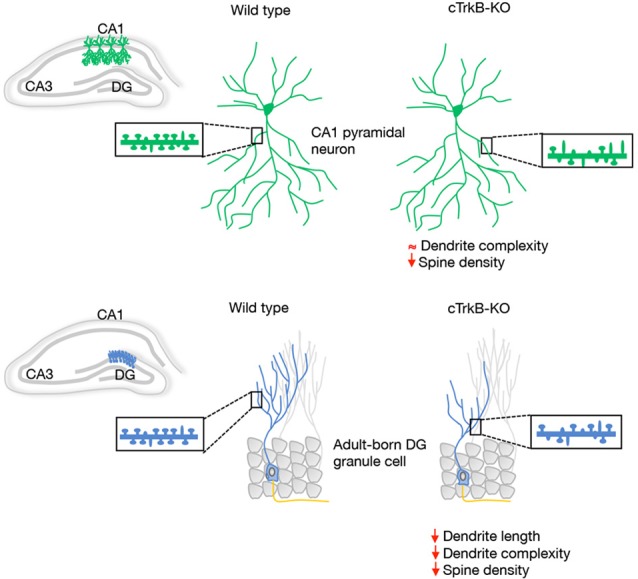
Schematic representation of the dendrite morphology and dendritic spine density reported *in vivo* in hippocampal CA1 pyramidal neurons and adult-born DG granule cells (GCs) from wild type and conditional tropomyosin receptor kinase B knockout (cTrkB-KO) mice. The insert displays examples of dendritic shafts showing an array of mushroom (mature) and thin (immature) dendritic spines.

A morphological correlate of synaptic plasticity is represented by the complexity of the dendritic arbors as well as by the density, shape and size of dendritic spines. The role of BDNF as a modulator of the dendritic structure of hippocampal pyramidal neurons *in vivo* is still unclear. However, a clear role for BDNF in hippocampal dendrite development has been observed in cultured pyramidal neurons (Cheung and Ip, 2007; Ji et al., 2010; Kwon et al., 2011; Lazo et al., 2013). Numerous studies have reported that BDNF increases dendritic spine density and induces long-term potentiation (LTP) in hippocampal pyramidal neurons. Consistently with this, TrkB-deficient mice have significantly fewer dendritic spines and excitatory synapses on CA1 neurons (Luikart et al., 2005; von Bohlen und Halbach et al., 2008; Figure 1).

In the adult DG, multiple studies reported a clear contribution of BDNF and TrkB in dendrite morphogenesis of newborn hippocampal neurons. The contribution of BDNF/TrkB signaling in the integration of newborn neurons was studied in conditional mice in which TrkB was deleted specifically in adult progenitors. In this study, the authors demonstrated that dendritic and spine growth of adult-born GCs is reduced in these animals (Figure 1). In line with this, a significant reduction in dendritic development, synaptic formation and maturation has been observed in postnatal-born granule neurons in different BDNF-mutant mice (Chan et al., 2008; Gao and Chen, 2009) and BDNF secreted by newborn GCs has been shown to function as an autocrine factor involved in dendrite development and synaptic maturation (Wang et al., 2015).

Since BDNF plays a critical role in the maintenance and refinement of neuronal circuits involved in learning and memory, diverse mechanisms are used to regulate its activity. In this review, we provide new insights into the mechanisms that regulate and diversify BDNF biology in hippocampal neurons, such as trafficking and subcellular compartmentalization of different *Bdnf* mRNA species, conversion of pro-BDNF to mature BDNF and BDNF-propeptide, modulation of BDNF signaling by novel TrkB receptor interactors and pre- vs. postsynaptic release of BDNF. Interestingly, these regulatory mechanisms allow BDNF to exert a rapid and dynamic refinement of the hippocampal connections in response to experience-dependent neuronal activity.

## Subcellular Compartmentalization of *Bdnf* mRNA Contributes to Synaptic Plasticity

**Figure 2:**
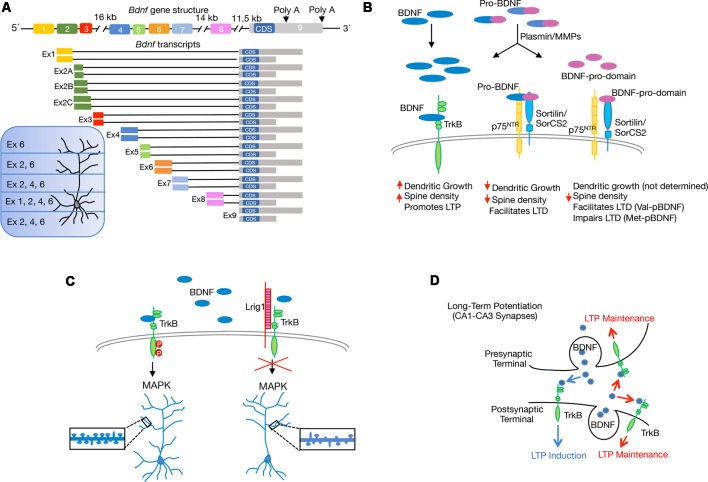
Scheme showing recent molecular and cellular mechanisms through which brain-derived neurotrophic factor (BDNF) regulates structural and functional synaptic plasticity in the hippocampus. **(A)** Schematic representation of rat *Bdnf* gene and splice variants derived from it, with different 5′untranslated region (UTR) and 3′UTR. The coding sequence (CDS) is indicated. Expression of BDNF generated by the different transcripts containing exons (Ex) is indicated according to Baj et al. (2011). **(B)** Illustration shows the extracellular proteolytic cleavage of pro-BDNF to give rise mature BDNF and the BDNF-prodomain, as well as the interaction of the different BDNF isoforms with specific receptors. The differences in the regulation of long-term depression (LTD) between the BDNF-prodomain polymorphisms: Val-pro-BDNF (Val-pBDNF) and Met-pro-BDNF (Met-pBDNF) are stated. **(C)** Model describing the endogenous inhibition of BDNF/TrkB signaling by the transmembrane protein Lrig1 and its implication for proximal dendrite development and spine formation in CA1-CA3 pyramidal neurons. **(D)** Model proposed for pre- vs. postsynaptic BDNF release and their contribution to long-term potentiation (LTP). Postsynaptic BDNF, as well as both presynaptic and postsynaptic TrkB, contribute to LTP maintenance (red arrows). Involvement of presynaptic BDNF and postsynaptic TrkB in LTP induction is also shown (blue arrows).

The subcellular compartmentalization of* Bdnf* mRNAs and its local secretion are necessary for proper development and plasticity. The synthesis of multiple transcripts is a mechanism that tightly controls BDNF expression. The rat *Bdnf* gene comprises nine exons but the coding sequence (CDS) resides in exon nine (Figure 2A). Thus, the eight upstream exons drive transcription of multiple *Bdnf* splice variants that encode an identical BDNF protein in a regional and cell-type specific manner (Liu et al., 2006; Aid et al., 2007). The presence of different *Bdnf* transcripts has led to propose the spatial code hypothesis, which suggests that differential expression of 5′ untranslated region (UTR) molecules allow spatial, temporal and stimulus-specific BDNF expression (Tongiorgi, 2008; Maynard et al., 2017). It is widely accepted that most *Bdnf* transcripts such as those containing exon 1 (Ex1) and Ex4, are found mainly within the cell body and proximal dendrites, whereas selected variants such as Ex2 and Ex6 are located in distal dendrites (Figure 2A). Thus, downregulation of *Bdnf* Ex1 and Ex4 transcripts in cultured hippocampal neurons reduces proximal dendrite number, while decreasing Ex2 and Ex6 transcripts alters distal dendrites (Baj et al., 2011). Moreover, mice with a selective disruption in individual *Bdnf* 5′UTR splice variants lead to local deficits in CA1 and CA3 dendrite and spine morphology (Maynard et al., 2017). Interestingly, the dysregulation of *Bdnf* transcripts containing specific 5′UTR exons has been associated with deficits in fear memory (Hill et al., 2016). The *Bdnf* gene also encodes two different 3′UTR, which add a new level of complexity to BDNF biology. The protein can be translated from different mRNA species containing either a short or a long 3′UTR (Timmusk et al., 1993; Figure 2A). While the short 3′UTR *Bdnf* mRNA is restricted to cell bodies in hippocampal neurons, the long 3′UTR* Bdnf* mRNA is transported to dendrites for local translation (An et al., 2008). Although BDNF abundance in dendrites is generally low, its dendritic localization is enhanced in response to depolarization (Tongiorgi et al., 1997), or to neurotrophin treatment (Righi et al., 2000; Vicario et al., 2015). Interestingly, *Bdnf*-mutant mice, carrying a truncated long 3′UTR, show impaired differentiation and maturation of adult-born hippocampal neurons (Waterhouse et al., 2012). By using long-term cultures of rat hippocampal neurons Orefice et al. (2013) suggested that somatically synthesized BDNF promotes spine formation, whereas dendritically synthesized BDNF is a key regulator of dendritic spine maturation. These findings indicate that the same protein synthesized in different neuronal compartments controls different aspects of the same biological process.

## Pro-BDNF and BDNF-Propeptides as Novel Regulators of Hippocampal Connectivity

Neurotrophins, and particularly BDNF, are initially synthesized as precursors, named proneurotrophins, in the endoplasmic reticulum, and can be converted into mature neurotrophins intracellularly by the action of furin or other proconvertases in the trans-golgi network or in secretory granules. Alternatively, these molecules can be secreted in their immature form, cleaved in the extracellular medium and converted into the mature form by plasmin and matrix metalloproteases (Lee et al., 2001). The efficiency of cleavage and the ratio of mature to proneurotrophins varies along different developmental stages (Yang et al., 2014; Hempstead, 2015). Proneurotrophins bind a complex composed by p75^NTR^ and either sortilin or SorCS2 (two members of the Vps10p-domain family). The p75^NTR^ receptor binds to the mature domain region or pro-BDNF whereas the prodomain binds to sortilin or SorCS2 (Teng et al., 2005; Anastasia et al., 2013). Increasing evidence indicates that mature and pro-BDNF exert opposing effects in the central nervous system (CNS). While mature BDNF promotes neuronal survival, differentiation, synaptic plasticity and LTP, the pro-BDNF induces apoptosis, growth cone retraction, reduces dendritic spine density and facilitates long-term depression (LTD) in hippocampal slices (Lu et al., 2005; Figure 2B).

Different studies have described secretion of both pro- and mature BDNF in response to depolarization (Nagappan et al., 2009; Yang et al., 2009; Je et al., 2012). In order to understand the physiological role of the pro-BDNF, Yang et al. (2014) generated a knock-in mouse in which the proconvertase/furin cleavage site of BDNF was mutated and expressed under the control of endogenous BDNF promoter. This work revealed that pro-BDNF secreted endogenously reduces dendrite arborization and spine density of hippocampal GCs *in vivo*. Deficits in p75^NTR^ rescue these dendritic defects, showing the requirement of p75^NTR^ as a mediator of pro-BDNF effects. Hippocampal slices from these pro-BDNF-expressing mice exhibit depressed synaptic activity and LTD. Interestingly, abnormalities in the ratio of pro-BDNF/mature BDNF has been described in the brain of individuals with autism, suggesting that the balance between these isoforms could be associated with the disease (Garcia et al., 2012).

The analysis of the human *Bdnf* gene revealed a single nucleotide polymorphism in the BDNF-prodomain which comprises a valine (Val) to methionine (Met) substitution at position 66 (Val66Met). This mutation disrupts the intracellular trafficking and activity-dependent release of BDNF (Chen et al., 2006) and has been associated to neuropsychiatric disorders (Egan et al., 2003; Soliman et al., 2010; Verhagen et al., 2010; Dincheva et al., 2012; Notaras et al., 2016).

Although for a long time, it was believed that the prodomain resultant from the cleavage of proneurotrophins was degraded after processing, later studies revealed that propeptides are present in brain tissue. Similarly to mature and pro-BDNF, BDNF-prodomain (pBDNF) is also secreted in an activity-dependent manner from hippocampal cultures, suggesting that it may act as an independent ligand (Goodman et al., 1996; Nagappan et al., 2009; Yang et al., 2009; Anastasia et al., 2013). The BDNF-prodomain has been shown to exert different neuronal activities depending on the presence of the 66Met substitution. Anastasia et al. (2013) reported that the Met amino acid induces structural changes within the BDNF-prodomain which allows Met-BDNF-prodomain (Met-pBDNF) to interact with SorCS2 and to trigger growth cone retraction in hippocampal neurons. Although the expression of p75^NTR^ is necessary for Met-pBDNF signaling, this ligand only interacts with SorCS2 (Figure 2C). Different groups analyzed the effect of BDNF-propeptide on dendritic spine development. Guo and collaborators tested the effects of the Val-pBDNF in cultured mature hippocampal neurons and observed that this ligand induces a reduction in dendritic spines density (Guo et al., 2016). In another study, it was shown that Met-pBDNF but not Val-pBDNF, can trigger disassembly of mature mushroom spines and synaptic contacts on cultured hippocampal pyramidal neurons exposed to these ligands during shorter periods of time (Giza et al., 2018). The discrepancy between the effects observed with Val-pBDNF could depend on the differences in the exposure time to the ligands used in the two studies. In agreement with the role of Met-pBDNF as a modulator of dendritic development, an *in vivo* assay shows that when the Met-pBNDF was delivered into the ventral CA1 region of the hippocampus, it resulted in a decrease in dendritic spine number and spine head size (Giza et al., 2018). In the same study, the authors showed that injection of the Met-pBDNF, but not Val-pBDNF display acute effects on circuitry and fear extinction behavior. Interestingly, the effects of Met-pBDNF were observed in a developmental period corresponding to the periadolescence, when the prodomain and its receptor are highly expressed in mice (Anastasia et al., 2013). Intriguingly, Mizui and collaborators demonstrated that the addition of Val-pBDNF facilitates LTD induction in hippocampal slices, while Met-pBDNF failed to induce LTD facilitation (Mizui et al., 2015), opening the question of how the different versions of the prodomain exert differential effects (Figure 2B). A possible answer is that each peptide triggers different signaling pathways.

## Control of BDNF Signaling by Novel Cell-Intrinsic TrkB Receptor Interactors

Genetically modified mouse models have established that TrkB receptors need to be modulated by different proteins to achieve cell-type-specific responses to its ligand during circuit development. Thus, members of the Vps10p-domain and leucine-rich repeat (LRR)-domain containing proteins have been described to be involved in the regulation of BDNF/TrkB signaling.

In addition, to act as a p75^NTR^ co-receptor required for pro-BDNF binding, SorCS2 was also identified as a physiological TrkB receptor interactor in hippocampal pyramidal neurons. SorCS2 is a type I transmembrane receptor that belongs to a Vps10p-domain family of sorting and signaling receptors which also includes Sortilin, SorLA and SorCS1 and 3 (Glerup et al., 2014). In contrast to the SorCS2/p75^NTR^ interaction, which is not influenced by electrical activity, high-frequency stimulation (HFS) promotes the binding of SorCS2 to TrkB. Notably, the interaction between TrkB and SorCS2 directs TrkB receptors to postsynaptic densities for synaptic tagging and contributes to LTP maintenance in a synapse-specific manner. Furthermore, hippocampal neurons lacking SorCS2 failed to induce TrkB phosphorylation and dendritic spine formation in response to BDNF. SorCS2-deficient mice display deficits in long-term memory and a higher tendency to take risk. Based on this evidence, it has been suggested that SorCS2 could be a possible link between proBDNF/BDNF signaling, synaptic plasticity (LTD/LTP) and mental disorders (Glerup et al., 2016). Interestingly, other members of the Vps10p-domain-containing protein, Sortilin, has been described as an interactor of ARHGAP33 involved in TrkB trafficking which is essential for synapse development (Nakazawa et al., 2016).

Previous evidence also demonstrated that engagement of TrkB with different LRR-domain-containing proteins (i.e., Slitrk5 and Lrig1) is a general mechanism that expands the repertoire of BDNF signaling outputs in specific population of neurons during nervous system development (Song et al., 2015; Alsina et al., 2016; Ledda and Paratcha, 2016). For instance, the LRR transmembrane protein Lrig1 was identified as a physiological regulator of proximal dendritic growth and BDNF function in CA1-CA3 pyramidal neurons. Deletion of Lrig1 mainly increases proximal complexity of apical dendritic arbors, revealing a novel molecular mechanism involved in the determination of basal vs. apical dendrite development. In line with this, overexpression of Lrig1 blocked dendritic spine formation induced by BDNF (Alsina et al., 2016; Figure 2C). The role of Lrig1 in the modulation of TrkB has also been analyzed in newborn neurons of the aged hippocampus. In the aging DG, newborn neurons remain immature for a long period of time, but voluntary exercise triggers their rapid growth and functional synaptic integration (Fan et al., 2017). Interestingly, Trinchero et al. (2017) demonstrated that plasticity of aged granule neurons is mediated in a cell-intrinsic manner by neurotrophin signaling. In this system, the knockdown of Lrig1, which promotes TrkB activation, accelerates neuronal development and potentiates the effects of running in newborn-GCs of aged mice. On the other hand, overexpression of Lrig1 in newly generated granule neurons of middle-aged mice abolished dendritic growth induced by running activity (Trinchero et al., 2017). Together, these findings reveal that physical exercise and neurotrophin signaling maximize plasticity of newborn GCs in the aged hippocampus. These data present physiological relevance since understanding the dynamics of experience-dependent connectivity remodeling throughout life is critical to prevent cognitive decline during aging and neurodegeneration (Mattson, 2012). Further research has extended this concept and showed that adult hippocampal neurogenesis combined with increased BDNF levels mimic the beneficial cognitive effects promoted by exercise in a mouse model of Alzheimer’s disease and suggest that enhancing neurogenesis and BDNF levels at early stages of the disease may protect against subsequent neuronal cell death (Choi et al., 2018).

## Pre vs. Postsynaptic Release of BDNF and Its Impact on LTP

Although it is clearly demonstrated that BDNF signaling regulates LTP in the hippocampus (Alder et al., 2005; Waterhouse and Xu, 2009; Panja and Bramham, 2014), the exact contribution of pre vs. postsynaptic BDNF secretion and TrkB signaling for synaptic plasticity remained controversial for several years. To address this, different approaches using genetic deletion of BDNF in specific hippocampal regions have been used (Zakharenko et al., 2003; An et al., 2008). In the last year, Lin et al. (2018) used a viral-mediated approach to delete BDNF or TrkB selectively in either the CA3 or CA1 region of the Schaffer collateral pathway to examine its impact for synaptic plasticity. These experiments revealed that presynaptic BDNF regulates the strength of LTP, while postsynaptic BDNF contributes to LTP maintenance. In addition, these experiments showed that LTP induction is mediated by anterograde BDNF/TrkB signaling, whereas both anterograde and retrograde BDNF/TrkB signaling are essential for LTP maintenance (Lin et al., 2018; Figure 2D). In a very elegant study using fluorescence resonance energy transfer (FRET)-based sensor for TrkB and two-photon fluorescent lifetime microscopy, Harward et al. (2016) identified an autocrine BDNF/TrkB signaling within a single stimulated dendritic spine of CA1 pyramidal neurons. In this study, the authors described a spine-autonomous autocrine signaling mechanism that evokes BDNF release from the same stimulated spine. The release of BDNF requires the activation of the NMDAR and CaMKII-dependent pathway. Subsequently, BDNF activates TrkB on the same stimulated spine to promote functional and structural plasticity. Therefore, these findings demonstrate that on one side, increased expression and secretion of BDNF is the result of stimulus-evoked neuronal activity and that on the other side, activity-evoked BDNF secretion strengthens synaptic potentiation and modulates axo-dendritic morphology in a local and synapse-specific manner. This work is in line with a previous study showing that synaptic stimulation of a single spine induces a gradual enlargement of spine heads that is dependent on protein synthesis and mediated by BDNF secretion (Tanaka et al., 2008). These studies provide new insight into the role of axonal and dendritic BDNF and TrkB receptor signaling in hippocampal LTP. Further behavioral experiments will be required to precisely understand the role of pre vs. postsynaptic BDNF/TrkB signaling in different learning and memory paradigms.

## Perspectives

The studies summarized here, describe multiple mechanisms that enable a rapid, localized and dynamic control of BDNF functions in hippocampal neurons. Impairment in these mechanisms leads to defects in synaptic plasticity and memory processes, which highlights their biological relevance.

Despite the recent progress in understanding the mechanisms underlying BDNF biology, many questions still remain to be answered. For instance, the role of pro-BDNF in the control of adult hippocampal neurogenesis has been barely addressed. Future studies undoubtedly are necessary to dissect the physiological contribution of the different BDNF splice variants as well as mature and immature forms of BDNF in specific synapses of the adult hippocampal circuit. For this, it will be important to determine the exact cellular source and the mechanism of biosynthesis and release of BDNF, pro-BDNF and BDNF-prodomain at specific synapses. Indeed, it will be also important to characterize the array of BDNF receptors, co-receptors and endogenous TrkB regulators present *in vivo* at specific synaptic connections of the hippocampus.

Dysfunction of the BDNF/TrkB system is involved in the onset of brain disorders, such as Alzheimer, autism and depression (Sungur et al., 2017; Numakawa et al., 2018). Therefore, understanding the basic biology of the different BDNF isoforms, receptors and interactors at the synapse will provide useful insights for the design of therapeutic tools for different neuropsychiatric diseases.

## Author Contributions

All the authors wrote the article and contributed with ideas and discussion of this topic.

## Conflict of Interest Statement

The authors declare that the research was conducted in the absence of any commercial or financial relationships that could be construed as a potential conflict of interest.
